# Microbial and inflammatory‐based salivary biomarkers of head and neck squamous cell carcinoma

**DOI:** 10.1002/cre2.139

**Published:** 2018-11-28

**Authors:** Anna Vesty, Kim Gear, Kristi Biswas, Fiona J. Radcliff, Michael W. Taylor, Richard G. Douglas

**Affiliations:** ^1^ Department of Surgery The University of Auckland New Zealand; ^2^ Otorhinolaryngology Auckland District Health Board New Zealand; ^3^ Department of Molecular Medicine & Pathology The University of Auckland New Zealand; ^4^ School of Biological Sciences The University of Auckland New Zealand; ^5^ Maurice Wilkins Centre for Molecular Biodiscovery The University of Auckland New Zealand

**Keywords:** cytokines, head and neck cancer, oral microbiome, oral mycobiome, saliva

## Abstract

Head and neck squamous cell carcinoma (HNSCC) patients often present with poor oral health, making it difficult to assess the relationship between oral microbes, inflammation, and carcinoma. This study investigates salivary microbes and inflammatory cytokines as biomarkers for HNSCC, with consideration of oral health. Saliva was collected from 30 participants, including 14 HNSCC patients and 16 participants representing both dentally compromised and healthy individuals. Bacterial and fungal communities were analyzed based on 16S rRNA gene and ITS1 amplicon sequencing, respectively, and concentrations of inflammatory cytokines were quantified using a cytometric bead array, with flow cytometry. Diversity‐based analyses revealed that the bacterial communities of HNSCC patients were significantly different to those of the healthy control group but not the dentally compromised patients. Fungal communities were dominated by *Candida*, irrespective of cohort, with Candida albicans comprising ≥96% of fungal sequences in most HNSCC patients. Significantly higher concentrations of interleukin (IL)‐1*β* and IL‐8 were detected in HNSCC and dentally compromised patients, when independently compared with healthy controls. IL‐1*β* and IL‐8 concentrations were significantly positively correlated with the abundance of C. albicans. Our findings suggest that salivary microbial and inflammatory biomarkers of HNSCC are influenced by oral health.

## 1 INTRODUCTION

Traditionally, the major risk factors associated with head and neck cancer are tobacco use and heavy alcohol consumption. More recently, viral infections—particularly infection with human papilloma virus subtype 16—have been implicated in the increasing incidence of this group of cancers, most notably in younger adults (Chaturvedi, Engels, Anderson, & Gillison, 2008). Emerging hypotheses suggest that oral microbial imbalances and variations to microbial community structure may modulate viral infections by regulating host susceptibility to oncogenic viruses (Vyshenska, Lam, Shulzhenko, & Morgun, 2017).

Salivary microbes are gaining attention as potential diagnostic markers for oral cancers. Recent studies suggest that increases in the relative abundances of several bacterial genera (including *Dialister*, *Selenomonas*, *Streptococcus*, and *Treponema*) occur in the saliva of oral cancer patients, when compared with healthy controls (Guerrero‐Preston et al., 2016; Pushalkar et al., 2011; Wolf, Moissl‐Eichinger, Perras, Koskinen, & Peter, 2017). Although Streptococcus anginosus has been implicated in head and neck squamous cell carcinoma (HNSCC; Morita et al., 2003; Tateda et al., 2000), a majority of the focus has been on *Porphyromonas gingivalis*, which displays oncogenic properties directly linked to oral carcinogenesis (Ha et al., 2015). Detection of *P. gingivalis* and other oral pathobionts in healthy subjects makes it difficult to attribute carcinogenic potential to specific bacteria (Katz, Onate, Pauley, Bhattacharyya, & Cha, 2011). Candida albicans is implicated in carcinogenesis because of its capacity to produce carcinogenic levels of acetaldehyde and induce host immune responses (Ramirez‐Garcia et al., 2016). Candidiasis may play a role in the malignant transformation of oral lesions (Bakri, Hussaini, Holmes, Cannon, & Rich, 2010). Furthermore, C. albicans is overrepresented (average relative abundance of 61.2%) in the mycobiome of oral squamous cell carcinoma (OSCC) biopsies (Perera et al., 2017).

Oral pathobionts elicit a host inflammatory response characterized by an increase in cytokines, chemokines, and growth factors that promote cell survival and proliferation, actions which may contribute to carcinogenesis (Cekici, Kantarci, Hasturk, & Van Dyke, 2014). Several studies report higher concentrations of inflammatory cytokines in the saliva of oral cancer patients, particularly interleukin (IL)‐1*β*, IL‐6, and IL‐8, relative to healthy controls (reviewed by Cheng, Rees, & Wright, 2014). It is unclear what induces these inflammatory‐based biases, but oral microbes may play a role.

Investigating oral and oropharyngeal carcinogenesis by studying oral microbiology or immunology is potentially confounded by oral health, given that HNSCC patients often present with poor oral health (Galvão‐Moreira & Da Cruz, 2016; Tezal et al., 2009). Poor oral health may be the factor that leads to differences in the microbiology and immune response seen between patients with HNSCC and healthy controls. In order to unmask oral health‐dependent bias and help decipher the link between HNSCC, oral microbes, and inflammation, we studied the oral bacterial and fungal communities in conjunction with salivary inflammatory markers in three disease groups: HNSCC patients, dentally compromised patients, and healthy controls.

## 2 METHODS

### 2.1 Ethics statement

Ethical approval for this study was granted by the Southern Health and Disability Ethics Committee (14/STH/121), and the study was conducted following the principles of the Declaration of Helsinki. Written informed consent was obtained from all participants.

### 2.2 Participant populations and sample collection

#### 2.2.1 HNSCC patients

Fourteen patients (11 males and 3 females, aged 49–81 years) diagnosed with HNSCC were recruited for this study during routine preradiotherapy dental assessments at the Oral Health Unit, Greenlane Hospital, Auckland, New Zealand. This group of patients included two current smokers, six ex‐smokers, five nonsmokers, and one patient for whom this information was not obtained. Dental and oral health information were extracted from clinical notes and orthopantomograms (Data S1).

#### 2.2.2 Dentally compromised patients

Nine patients (seven male and two female, aged 28–68 years) who attended Relief of Pain Clinics at the Oral Health Unit, Greenlane Hospital, Auckland, New Zealand were recruited to participate in this study. Two patients were current smokers, three ex‐smokers, and four nonsmokers. Based on the World Health Organization definition of oral health (World Health Organization, 2012), this group of patients was deemed dentally compromised as the result of periodontal disease and/or tooth decay that caused significant pain and necessitated tooth extraction at this clinic. Samples were collected prior to clinical intervention.

#### 2.2.3 Healthy controls

Seven volunteers from The University of Auckland, New Zealand participated in this study representing healthy controls. To maintain anonymity, participant demographics including age and sex were not assigned to samples, however, participants were nonsmokers and aged approximately 20–35 years at the time of sample collection.

#### 2.2.4 Sample collection and storage

Approximately 1 ml of unstimulated whole saliva was collected in a sterile container from each participant (~between 9:00 a.m.–1:00 p.m.) and divided into 200 μl aliquots. All samples were frozen at −20°C until further processing.

### 2.3 DNA extraction, amplification, and sequencing preparation

Genomic DNA (gDNA) was extracted using a phenol:chloroform‐based DNA isolation method we have previously described (Vesty, Biswas, Taylor, Gear, & Douglas, 2017). PCR‐grade water was used as an extraction control. The V3–V4 region of the bacterial 16S ribosomal RNA gene was amplified using primers 341F and 806R (Klindworth et al., 2013), both with Illumina compatible overhang adapter sequences. Each PCR reaction contained HotStar PCR Buffer (1×), 2 mM MgCl_2_, 0.5 mM of each dNTP, 0.5 U HotStar DNA Polymerase (Qiagen, Hilden, NRW, Germany), 0.2 μm of each primer, 1 μl of gDNA template and PCR‐grade water to a final volume of 25 μl. Positive and negative PCR controls were included (Escherichia coli and water, respectively). PCR was performed using the following conditions: Initial denaturation at 95°C for 15 min, followed by 32 cycles of denaturation (95°C for 30 s), annealing (55°C for 30 s), and extension (70°C for 40 s), with a final extension step at 70°C for 3 min. For the fungal PCR reactions, Illumina‐compatible primers ITS1F (Gardes & Bruns, 1993) and ITS2 (White, Bruns, Lee, & Taylor, 1990) were substituted in to amplify the internal transcribed spacer 1 (ITS1) region of fungal DNA. *C*. *albicans* was used as a positive control and water as a negative control. Fungal PCR conditions deviated from those described for bacteria as follows: annealing temperature was reduced to 50°C and thermocycling increased to 38 cycles to account for expected lower fungal biomass (Vesty et al., 2017). Duplicate PCR reactions were prepared for sequencing as previously described (Vesty et al., 2017). Samples were sequenced using the Illumina MiSeq platform, through New Zealand Genomics Ltd. Sequencing data analyzed during this study are available from the NCBI SRA, uploaded under accession number SRP126472.

### 2.4 Processing of bacterial 16S rRNA gene sequence data

Using USEARCH (version 9.2), sequences were merged and filtered to remove poor‐quality sequences, singletons, and sequences <350 base pairs (bp). Operational taxonomic units (OTUs) were defined based on 97% 16S rRNA gene sequence similarity and simultaneously checked for chimeras within USEARCH. A second chimera check was performed against the Human Oral Microbiome Database (HOMD). Taxonomic assignment was performed in QIIME (version 1.9) through the Ribosomal Database Project classifier using the HOMD. Sequences were rarefied to 1,996 reads per sample for subsequent analyses.

### 2.5 Processing of fungal ITS1 sequence data

Fungal sequences were processed using a similar USEARCH pipeline, however, data were filtered to remove sequences <100 bp, and rare fungal sequences were filtered out using an abundance threshold of four sequences. OTUs were defined based on 97% sequence similarity and simultaneously checked for chimeras within USEARCH, followed by a second chimera check using UNITE as a reference. Taxonomic assignment was performed in QIIME using the BLAST method against UNITE (version 7.0). Nonfungal‐derived sequences were removed before subsampling to 229 reads per sample.

### 2.6 Estimation of diversity metrics

Alpha diversity metrics and Bray–Curtis dissimilarity were estimated independently for bacterial and fungal communities in QIIME (Caporaso et al., 2010). Beta diversity was visualized in R (version 3.4.2) based on the Bray–Curtis dissimilarity through nonmetric multidimensional scaling plots, generated with a maximum of 999 restarts (R Core Team, 2017).

### 2.7 Cytometric bead array

Thawed saliva was diluted 1:1 with phosphate buffered saline and prepared for flow cytometry (according to manufacturer's instructions) using the BD™ Cytometric Bead Array Human Inflammatory Cytokine Kit (BD Biosciences, Franklin Lakes, NJ, USA) to quantitatively measure IL‐8, IL‐1*β*, IL‐6, IL‐10, IL‐12p70, and tumor necrosis factor. Samples were acquired on an LSR II using FACSDiva™ Software v6.1.1 (BD Biosciences, Franklin Lakes, NJ, USA) and sample concentrations interpolated from standard curves for each cytokine using the radioimmunoassay option in GraphPad Prism v7.03.

### 2.8 Statistical analyses

Bacterial and fungal community data were compared independently for each group. The relative abundance of individual taxon‐assigned OTUs was statistically assessed using paired, two‐tailed *t* tests with the Bonferroni adjustment for multiple pairwise comparisons, with a significance value of 0.05. Alpha diversity metrics were assessed using a nonparametric *t* test. Bray–Curtis distance matrices were used to statistically assess beta diversity with pairwise, permutational multivariate analysis of variance (PERMANOVA) in PRIMER v6 software, using Type III (partial) sum of squares with unrestricted permutation of raw data and 999 permutations. Homogeneity of dispersions was calculated using PERMDISP in PRIMER v6 based on pairwise comparisons of deviations from the median with 999 permutations. Contribution of disease group to partitioning of Bray–Curtis distance matrices was assessed using the *Adonis* function in the Vegan package in R, with 999 permutations.

Linear Discriminant Analysis (LDA) Effect Size (LEfSe) was used to identify bacterial genera that differentiated the groups based on rarefied relative abundance data (Segata et al., 2011). The nonparametric factorial Kruskal–Wallis rank‐sum test (*α* ≤ 0.05) with a subsequent (unpaired) Wilcoxon rank‐sum test (*α* ≤ 0.05) and an all‐against‐one multiclass analysis were used; only genera reaching an LDA score threshold of ≥3 were reported.

IL‐1*β* and IL‐8 concentrations were log transformed to produce normally distributed data and excluded if the relevant cytokine concentration was below the limits of detection. IL‐1*β* and IL‐8 were compared between the three disease groups using a two‐way ANOVA and Tukey's post‐hoc test for multiple comparisons of means, with a 95% family‐wise confidence interval. Spearman's rank correlation coefficients (*ρ*) and corresponding *p* values were calculated with a null hypothesis in R to determine the statistical dependence between IL‐1*β* and IL‐8 concentrations and the relative abundance of the most abundant 30‐bacterial and five‐fungal genera. *Treponema* was also included in this analysis due to its potential clinical relevance, and *Candida* was included at OTU level. Significant correlations (*p* < 0.01) were visualized in R using corrplot, with hierarchical clustering (Wei & Simko, 2017).

## 3 RESULTS

After postprocessing and filtering, 533,317 bacterial 16S rRNA gene sequences were obtained, yielding 173 OTUs, classified into 68 genera. Fungal data returned 1,036,947 sequences of which 8,435 (0.8%) were nonfungal derived and therefore removed. Of the 1,028,512 fungal sequences that were retained, 44 OTUs from 34 genera were detected. Rarefaction curves based on the “observed species” metric indicated that sequencing depth was sufficient to capture the vast majority of bacterial and fungal diversity (Data S2).

### 3.1 Bacterial community profiles

**Figure 1:**
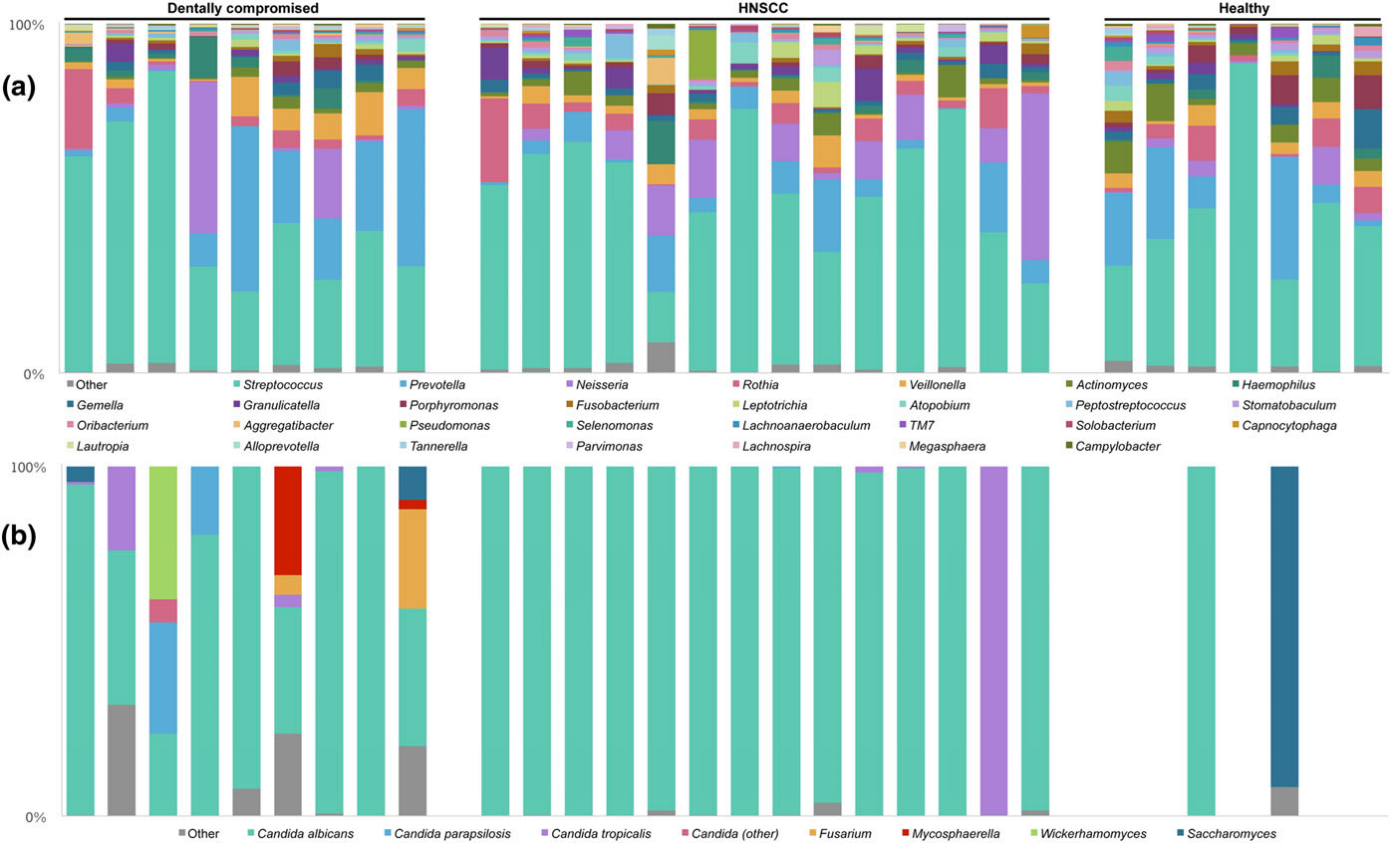
Genus‐level summary of microbial communities in saliva by disease group. Each bar in (a) reflects the bacterial community in one sample and is aligned with its corresponding fungal community in (b). (a) 30 most abundant bacterial genera (on average) rarefied to 1996 sequences per sample; and (b) five most abundant (on average) fungal genera, with *Candida*‐assigned sequences identified at operational taxonomic unit level, excluding five healthy control samples that did not meet the fungal rarefication criterion

Bacterial communities were largely dominated by *Streptococcus*, which accounted for an average of 47% of sequences across the three groups. *Prevotella*, *Neisseria*, *Rothia*, and *Veillonella* were also dominant contributors to the bacterial communities in all groups, representing an average sequence abundance of 12%, 8%, 5% and 4%, respectively (Figure 1a). Pairwise comparisons of the relative abundance of individual taxon‐assigned OTUs identified no significant differences between the three disease groups. LEfSe analyses indicated that the genus *Treponema* was associated with the dentally compromised patients, whereas *Actinomyces* and *Fusobacterium* were associated with the healthy controls (Data S3). No genus was significantly associated with HNSCC patients.

### 3.2 Bacterial diversity

**Figure 2:**
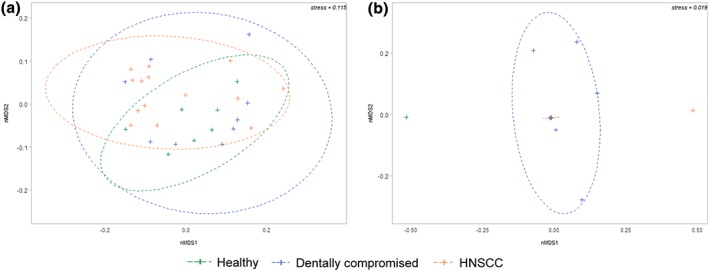
Bray–Curtis dissimilarity nMDS plots by disease group depicting (a) bacterial beta diversity and (b) fungal beta diversity. Ellipses represent a 95% confidence interval for each disease group. Data that failed subsampling thresholds were excluded, therefore, no ellipse was calculated for healthy controls (*n* = 2) in (b)

Pairwise comparisons of bacterial alpha diversity metrics failed to identify any significant differences between groups, although the number of observed species in the dentally compromised group was slightly higher at 63.7 ± 14.6, compared with the HNSCC (56.8 ± 14.4) and healthy control (54.4 ± 20.9) groups (mean ± SD). Based on Bray–Curtis dissimilarity, disease group accounted for approximately 11% of the variation in bacterial data. PERMANOVA indicated that a significant proportion of bacterial community variation was attributable to differences between the HNSCC patients and the healthy control group (*p* = 0.03, Figure 2a). No other significant differences were identified in remaining pairwise PERMANOVA comparisons. Multivariate dispersions were compared based on the distance of each sample to the median of their disease group. The dentally compromised patients (0.37 ± 0.04) had the greatest average deviations from the median (mean ± SE). However, pairwise comparisons between disease groups revealed no significant differences, with deviations of 0.34 ± 0.03 for HNSCC patients and 0.32 ± 0.03 for healthy controls.

### 3.3 Fungal community profiles

Five of the seven healthy controls were excluded from fungal community analyses due to insufficient sequence reads, which probably reflects an absence or comparatively lower abundance of fungi in this group. There was no consistency between the two healthy control samples included in the fungal analysis, with one dominated by C. albicans and the other by *Saccharomyces* (Figure 1b). The remaining samples from all disease groups were largely dominated by *Candida* (Figure 1b). NCBI BLAST checks of OTUs belonging to the *Candida* genus revealed C. albicans (100% sequence identity) comprised on average about 80% of obtained sequence reads across all disease groups. However, fungal communities in HNSCC patients were almost entirely since of C. albicans (individual relative abundances of 96–100%). There was one exception where the mycobiome comprised 99.6% Candida tropicalis (NCBI BLAST 100% sequence identity), however, no obvious characteristic differentiated this HNSCC patient from the rest.

### 3.4 Fungal diversity

Dentally compromised patients exhibited the most diverse fungal communities, which included sequences belonging to the fungal genera *Candida*, *Fusarium*, *Mycosphaerella*, *Saccharomyces*, and *Wickerhamomyces* (Figure 1b). This finding was reflected in alpha diversity metrics, with significantly more fungal observed species found in the dentally compromised patients (5.3 ± 3.8) than in HNSCC patients (1.7 ± 0.5; *p* = 0.003). Only two samples from the healthy control group, which reached the rarefication threshold, were included in this analysis (1.7 ± 0.7).

Disease group also significantly contributed to variance in fungal communities (*p* = 0.03), explaining approximately 19% of observed mycobiota variation. PERMANOVA revealed that disease group was a significant driver of fungal community structure, with a significant difference identified between the HNSCC and dentally compromised patients (*p* = 0.04, Figure 2b). This result was supported by a pairwise comparison of dispersions based on deviations from the median for each disease group, with a difference trending towards significance between the HNSCC and dentally compromised patients (*p* = 0.06).

### 3.5 Comparison of inflammatory cytokine concentrations

Three of the six tested inflammatory cytokines were detectable in saliva samples: IL‐1*β*, IL‐6, and IL‐8. Across all groups, IL‐8 was the most frequently detected, found in 28 of the 30 subjects (93%), followed by IL‐1*β* in 26 subjects (87%); IL‐6 was only detected in seven of the 30 subjects (23%), six of whom were HNSCC patients (Table 1). Comparisons for each inflammatory cytokine revealed the concentration of IL‐1*β* was significantly higher in the HNSCC and dentally compromised patients, when these two disease groups were individually compared with the healthy controls (Table 2). The concentration of IL‐1*β* was 5.1 times higher in the HNSCC patients when compared with healthy controls, yet no significant differences were found when the HNSCC patients were compared with the dentally compromised group. Comparison of IL‐8 concentrations produced similar results: IL‐8 was significantly higher (6.7 times) in the HNSCC patients compared with healthy controls and 6.5 times higher in the dentally compromised patients compared with healthy controls (Table 2). No significant difference in IL‐8 concentration was detected between the HNSCC and the dentally compromised patients (Table 2).

**Table 1:** Summary of detectable inflammatory cytokines

	HNSCC	Dentally compromised	Healthy
IL‐1*β* a	11/14 (79%)	9/9 (100%)	6/7 (86%)
Concentration IL‐1*β* b	5.1 ± 0.9	5.2 ± 1.5	3.5 ± 0.9
IL‐6^a^	6/14 (43%)	1/9 (11%)	0/7 (0%)
Concentration IL‐6^b^	−0.8 ± 1.2	−0.1 ± N/A	N/A
IL‐8^a^	13/14 (93%)	9/9 (100%)	6/7 (86%)
Concentration IL‐8^b^	7.2 ± 1.4	7.1 ± 1.2	5.3 ± 2.0

a Number of patients cytokine detected in/total patients in disease group.

b Cytokine concentration (log pg/ml; mean ± SD).

**Table 2:** Two‐way ANOVA summary of IL‐1*β* and IL‐8 concentration comparisons

	HNSCC versus healthy	Dentally compromised versus healthy	HNSCC versus dentally compromised
Difference IL‐1*β* a	5.1	5.4	0.93
*p* value^b^	**0.03**	**0.02**	**0.99**
Difference IL‐8^a^	6.7	6.5	1.0
*p* value^b^	**0.04**	**0.05**	**1.00**

a Fold difference in cytokine concentration A to B (A vs. B).

b Significant *p* values expressed in bold (*α* = 0.05). Tukey's post‐hoc test, 95% confidence interval.

### 3.6 Correlation of microbial relative abundance data to IL‐1*β* and IL‐8 concentrations

**Figure 3:**
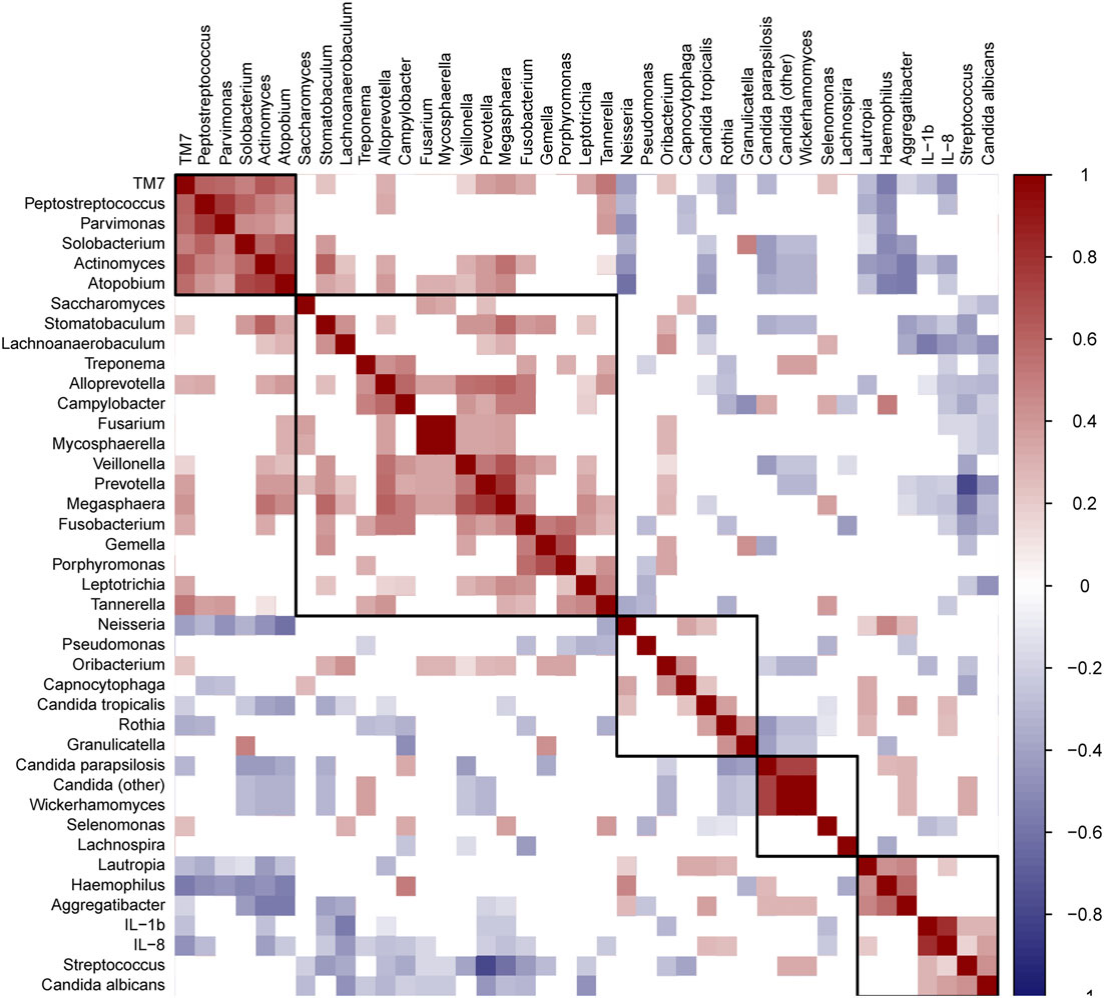
Correlation matrix visualizing significant correlations (*p* < 0.01) between microbial relative abundance data and the concentrations of IL‐1*β* and IL‐8. Positive correlations are visualized in shades of red and negative correlations in shades of blue; correlations where the *p* value was ≥0.01 are left blank. Correlations are ordered by hierarchical clustering, with clusters outlined

Calculation of Spearman's rank correlation coefficients (*ρ*) comparing IL‐1*β* and IL‐8 with the 30 most abundant bacterial genera, plus *Treponema*, and the five most abundant fungal genera (*Candida*‐assigned sequences at OTU level) returned *ρ* values ranging from −0.56 to 0.37. The strongest positive correlation occurred between IL‐8 and C. albicans (*ρ* = 0.37, *p* = < 0.001); IL‐1*β* also showed significant positive correlation with the relative abundance of C. albicans (*ρ* = 0.30, *p* = < 0.001). Positive correlations between bacterial genera and IL‐1*β* and IL‐8 concentrations returned *ρ* values of ≤0.30, suggesting only weak correlations, as summarized in Figure 3. The relative abundance of the periodontopathogenic genera *Porphyromonas*, *Tannerella*, and *Treponema* showed no significant positive correlations with cytokine concentrations. However, the relative abundances of these genera were significantly positively correlated with each other (Figure 3).

Several bacterial genera were negatively correlated with IL‐1*β* and IL‐8, with the strongest negative correlations occurring between IL‐1*β* and *Lachnoanaerobaculum* (*ρ* = −0.56, *p* = < 0.001) and *Actinomyces* and IL‐8 (*ρ* = −0.42, *p* < 0.001). Eight bacterial genera were significantly negatively correlated with both IL‐1*β* and IL‐8 (*p* < 0.01): *Actinomyces*, *Alloprevotella*, *Lachnoanaerobaculum*, *Megasphaera*, *Prevotella*, *Selenomonas*, *Stomatobaculum*, and a genus within the candidate phylum TM7. Multiple significant correlations occurred between the relative abundances of individual microbial genera (summarized in Figure 3) and notably included a strong negative correlation between *Streptococcus* and *Prevotella*, two genera that dominated the salivary bacterial communities (*ρ* = −0.80, *p* = < 0.001).

## 4 DISCUSSION

The role of oral microbes in the pathogenesis of HNSCC is not well understood. Generally, it is considered that oral microbes contribute to carcinogenesis, potentially accounting for differences observed in the oral microbial communities of head and neck cancer patients. However, it could also be considered that oral microbial communities might be modified as a *consequence* of the cancer. Saliva‐based microbiome studies have not yielded a consensus but do suggest that overall bacterial community composition, rather than the presence of a single pathogen, may be important in head and neck cancer. However, such conclusions can be confounded by the choice of control group and may simply reflect oral health‐related differences rather than differences related to cancer per se. To clarify this issue, a comparison with a group of dentally compromised patients in addition to healthy controls was included in this study.

PERMANOVA analysis of beta diversity metrics generated in this study indicated that there was a significant difference in salivary bacterial community structure between healthy controls and HNSCC patients. However, no significant variations were detected in the bacterial beta diversity profiles of the HNSCC and dentally compromised patients, suggesting similarity in the bacterial communities of these two patient groups. Based on genus‐level LEfSe analysis, HNSCC patients had no distinguishing bacterial characteristics.

Although several studies have focused on deciphering the bacterial features of oral cancers, far less attention has been devoted to the oral mycobiome. We found that C. albicans comprised 96–100% of fungal sequences in the saliva of most HNSCC patients, compared with an average of 80% of the mycobiome of all subjects reaching the rarefication threshold. The significantly more diverse mycobiome in dentally compromised patients highlights the overrepresentation of C. albicans in HNSCC patients. C. albicans has been recognized as an etiological factor in oral carcinogenesis due to its ability to induce host inflammatory responses (Ramirez‐Garcia et al., 2016). This observation was supported by our finding that the relative abundance of C. albicans was positively correlated with the concentrations of IL‐1*β* and IL‐8 in saliva. However, the correlations between C. albicans and IL‐1*β* and IL‐8 were not particularly strong, potentially reflecting the presence of this OTU in most subjects.

Detection of IL‐6 was most frequently observed in the HNSCC group, and this cytokine has previously been implicated as a potential salivary biomarker of OSCC (Cheng et al., 2014). In vitro, IL‐6‐induced inflammation promotes tumorigenesis in oral cancer cells via aberrant DNA methylation (Gasche, Hoffmann, Boland, & Goel, 2011). IL‐1*β* and IL‐8 inflammatory profiles of HNSCC patients suggest the presence of comparable levels of inflammation to dentally compromised patients, with concentrations significantly higher in both of these patient groups when compared independently with healthy controls. Smoking has been linked to lower concentrations of IL‐1*β*, IL‐6, and IL‐8 in saliva (Rathnayake et al., 2013), and although the healthy control group in this study was composed only of nonsmokers, this group still had the lowest average salivary concentrations of these cytokines. The presence of smokers/ex‐smokers in the HNSCC and dentally compromised groups (and absence in the healthy controls) may reflect the epidemiology of head and neck cancer and periodontal disease, and that tobacco use is a strong risk factor for both (Petersen & Ogawa, 2012; Sturgis, Wei, & Spitz, 2004). Periodontal disease is linked with higher salivary concentrations of IL‐1*β* and IL‐8 (reviewed by Jaedicke, Preshaw, & Taylor, 2016), yet in this study, it was unclear whether inflammatory profiles were driven by differences in the respective microbial communities, with only weak associations detected between microbial relative abundances and cytokine concentrations.

The results of this study suggest that the use of salivary bacterial communities as a biomarker of HNSCC is limited due to its reduced distinguishability from dentally compromised patients. Similarly, differentiation of HNSCC and dentally compromised patients based on inflammatory cytokines is limited, with only healthy controls significantly differentiated. However, application of IL‐6 as a biomarker for HNSCC is potentially relevant due to its increased detection in HNSCC patients, although this finding requires confirmation with a larger cohort. The elevated relative abundance of C. albicans as a constituent of the salivary mycobiome is confounded by its presence in both healthy controls and dentally compromised patients. Finally, this study highlights the importance of considering oral health when attempting to discern microbial and inflammatory biomarkers of HNSCC in saliva.

## CONFLICT OF INTEREST

None to declare.

## Supporting information


**Data S1.** HNSCC patient information

**Data S2.** Alpha rarefaction analysis plots demonstrating the number of ‘observed species’ in saliva by disease group as a function of sequences per sample for (a) bacterial observed species and (b) fungal observed species.

**Data S3.** Differential relative abundances of bacterial genera associated with disease class through LEfSe analysis: (a) *Actinomyces*; (b) *Fusobacterium*; (c) *Treponema*. Mean and median relative abundances for each disease class are indicated by the solid and dashed lines, respectively.
