# Supplementary material for: Microbial and inflammatory‐based salivary biomarkers of head and neck squamous cell carcinoma
Source: Clin Exp Dent Res. 2018 Nov 28;4(6):255–62. doi: 10.1002/cre2.139 (PMC6305924; doi:10.1002/cre2.139)
Supplement: Supplementary file 1 — Data S1. HNSCC patient information [file CRE2-4-255-s001.docx]

**Supplementary 1. HNSCC patient information**

| Patient | Tumour location | ^a^DMFT index | | | Dentures | ^b^Periodontal disease | ^c^Pre-RT extraction |
| --- | --- | --- | --- | --- | --- | --- | --- |
|  |  | **D** | **M** | **F** |  |  |  |
| 1 | left parotid | 0 | 10 | 16 | N |  | N |
| 2 | buccal mucosa | 1 | 6 | 11 | N | Y | Y |
| 3 | right tongue | 0 | 4 | 15 | N |  | N |
| 4 | left palate | 0 | 7 | 5 | N |  | N |
| 5 | left parotid | 0 | 26 | 0 | N |  | N |
| 6 | floor of mouth | 1 | 5 | 6 | N |  | N |
| 7 | lateral tongue | 4 | 19 | 10 | N |  | Y |
| 8 | floor of mouth | 6 | 12 | 13 | N | Y | Y |
| 9 | left tonsil | 1 | 11 | 6 | N |  | Y |
| 10 | base of tongue | 0 | 17 | 0 | Y | Y | Y |
| 11 | left tonsil | 0 | 9 | 20 | N |  | N |
| 12 | left tonsil | 2 | 12 | 14 | N |  | Y |
| 13 | base of tongue | 0 | 11 | 13 | N |  | N |
| 14 | left tonsil | 0 | 6 | 21 | N |  | N |

^a^ *Decayed (D), Missing (M), Filled (F) Teeth (DMFT) index describing number of affected teeth, based on orthopantomograms*

^b^ *Evidence of periodontal disease, based on orthopantomograms*

^c^ *Pre-radiotherapy extraction/s of compromised teeth required*
