# Supplementary material for: Microbial and inflammatory‐based salivary biomarkers of head and neck squamous cell carcinoma
Source: Clin Exp Dent Res. 2018 Nov 28;4(6):255–62. doi: 10.1002/cre2.139 (PMC6305924; doi:10.1002/cre2.139)
Supplement: Supplementary file 2 — Data S2. Alpha rarefaction analysis plots demonstrating the number of ‘observed species’ in saliva by disease group as a function of sequences per sample for (a) bacterial observed species and (b) fungal observed species. [file CRE2-4-255-s002.pdf]

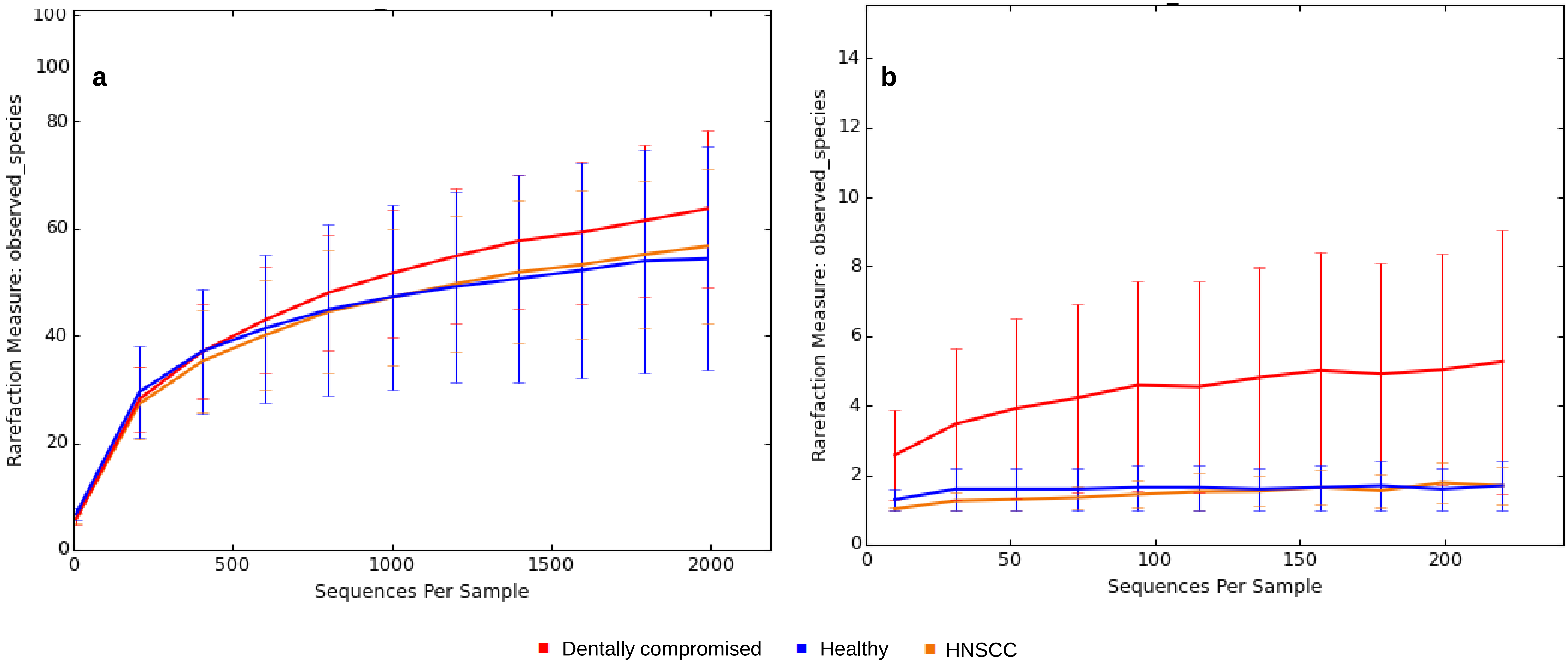

Supplementary 2. Alpha rarefaction analysis plots demonstrating the number of ‘observed species’ in saliva by disease group as a function of sequences per sample for (a) bacterial observed species and (b) fungal observed species.
