# Supplementary material for: Microbial and inflammatory‐based salivary biomarkers of head and neck squamous cell carcinoma
Source: Clin Exp Dent Res. 2018 Nov 28;4(6):255–62. doi: 10.1002/cre2.139 (PMC6305924; doi:10.1002/cre2.139)
Supplement: Supplementary file 3 — Data S3. Differential relative abundances of bacterial genera associated with disease class through LEfSe analysis: (a) Actinomyces; (b) Fusobacterium; (c) Treponema. Mean and median relative abundances for each disease class are indicated by the solid and dashed lines, respectively. [file CRE2-4-255-s003.pdf]

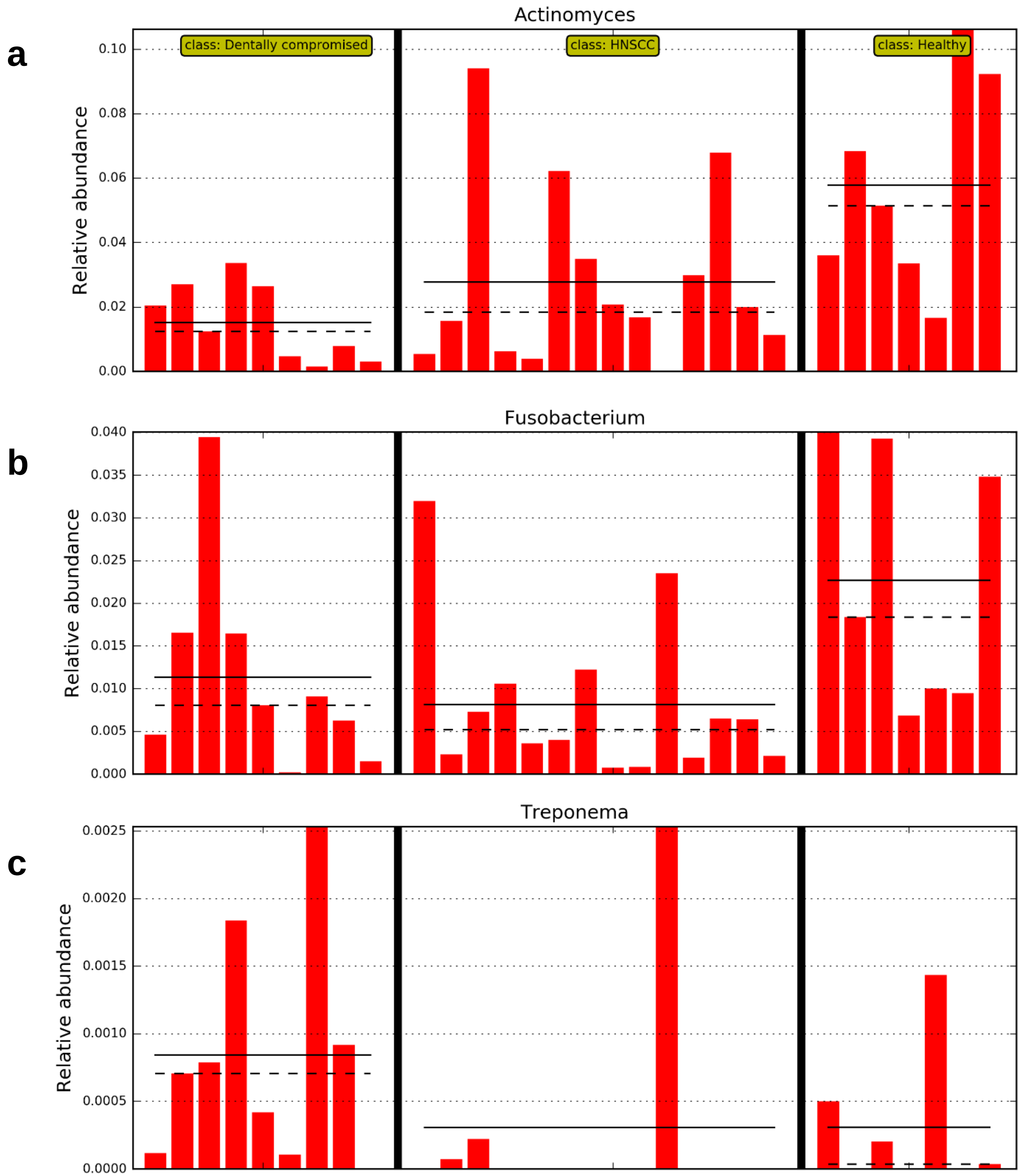

Supplementary 3. Differential relative abundances of bacterial genera associated with disease class through LEfSe analysis: (a) *Actinomyces*; (b) *Fusobacterium*; (c) *Treponema*. Mean and median relative abundances for each disease class are indicated by the solid and dashed lines, respectively.
